# The Role of MMP7 and Its Cross-Talk with the FAS/FASL System during the Acquisition of Chemoresistance to Oxaliplatin

**DOI:** 10.1371/journal.pone.0004728

**Published:** 2009-03-06

**Authors:** Vanessa Almendro, Elisabet Ametller, Susana García-Recio, Olga Collazo, Ignasi Casas, Josep M. Augé, Joan Maurel, Pedro Gascón

**Affiliations:** 1 Medical Oncology, Institut d'Investigacions Biomèdiques Agustí Pi y Sunyer (IDIBAPS), Institut Clínic de Malalties Hemato-Oncològiques (ICMHO), Hospital Clínic, Facultat de Medicina, Universitat de Barcelona, Barcelona, Spain; 2 Biochemical Department, Institut d'Investigacions Biomèdiques Agustí Pi y Sunyer (IDIBAPS), Institut Clínic de Malalties Hemato-Oncològiques (ICMHO), Hospital Clínic, Facultat de Medicina, Universitat de Barcelona, Barcelona, Spain; City of Hope Medical Center, United States of America

## Abstract

**Background:**

The efficacy of oxaliplatin in cancer chemotherapy is limited by the development of drug resistance. MMP7 has been related to the loss of tumor cell response to cytotoxic agents although the exact mechanism is not fully understood. Moreover, MMP7 is an independent prognosis factor for survival in patients with colorectal cancer. The aim of the present study was to analyze the role of MMP7 and its cross-talk with the Fas/FasL system during the acquisition of oxaliplatin resistance in colon cancer cells.

**Principal Findings:**

For this purpose we have developed three different oxaliplatin-resistant cell lines (RHT29, RHCT116 p53^+/+^, RHCT116 p53^−/−^) from the parental HT29, HCT116 p53^+/+^ and HCT116 p53^−/−^ colon cancer cells. MMP7 basal expression was higher in the resistant compared to the parental cell lines. MMP7 was also upregulated by oxaliplatin in both HT29 (p53 mutant) and RHCT116 p53^−/−^ but not in the RHCT116 p53^+/+^. Inhibition of MMP by 1,10-phenantroline monohydrate or siRNA of MMP7 restores cell sensitivity to oxaliplatin-induced apoptosis in both HT29 and RHCT116 p53^−/−^ but not in the RHCT116 p53^+/+^. Some of these effects are caused by alterations in Fas receptor. Fas is upregulated by oxaliplatin in colon cancer cells, however the RHT29 cells treated with oxaliplatin showed a 3.8-fold lower Fas expression at the cell surface than the HT29 cells. Decrease of Fas at the plasma membrane seems to be caused by MMP7 since its inhibition restores Fas levels. Moreover, functional analysis of Fas demonstrates that this receptor was less potent in inducing apoptosis in RHT29 cells and that its activation induces MAPK signaling in resistant cells.

**Conclusions:**

Taking together, these results suggest that MMP7 is related to the acquisition of oxaliplatin-resistance and that its inhibition restores drug sensitivity by increasing Fas receptor. Furthermore, Fas undergoes a change in its functionality in oxaliplatin-resistant cells inducing survival pathways instead of apoptotic signals.

## Introduction

Oxaliplatin has shown excellent efficacy in the treatment of colorectal cancer in combination with 5-fluorouracil. Despite its proven activity, the acquisition of drug resistance remains a major problem in patient management, ultimately leading to patient death [1]. Mechanisms of resistance to platinum agents such as oxaliplatin include increased DNA repair, overexpression of copper transporters, enhanced drug detoxification and increased tolerance for DNA damage [2], [3]. However, despite the fact that the mechanisms influencing treatment responses are well known, it appears that the major process leading to chemotherapy resistance [4] is the ability of cancer cells to evade cell death signals. This loss of response to apoptosis-induction during development of drug resistance resembles the normal tumor progression process, in which malignant cells also undergo molecular changes providing them with mechanisms against cell death induction [5], [6].

Fas (APO-1/CD95), a 48 kDa membrane protein belonging to the TNF receptor superfamily, activates caspase-dependent apoptosis in susceptible cells when is activated by its natural ligand (FasL). Many cancer cells acquire survival advantage during tumor progression by decreasing its sensitivity to Fas-induced apoptosis [6], [7]. Some mechanisms affecting Fas sensitivity include downregulation of Fas protein expression [8], and blockade of the active receptor site by the soluble form of Fas ligand (sFasL) [4], or by both. Matrix metalloproteinases (MMPs) are also implicated in the survival advantage of malignant cells influencing the Fas/FasL pathway.

MMPs are zinc-dependent enzymes associated with many types and stages of cancer. These enzymes promote metastasis and tumor growth through a variety of mechanisms such as ECM degradation, regulation of angiogenesis and modulation of innate immunity [9]. As a result of MMP activity tumor cells can develop mechanisms to evade immune responses, resulting in promotion of tumor survival, acquisition of metastatic phenotype and further tumor dissemination [10]. MMP7 (matrilysin), is a metalloproteinase with prometastatic function related to: 1) early tumor development [11], 2) metastatic potential [12] and 3) clinical outcome in cancer [13], [14]. We have observed in patients with advanced colorectal cancer that MMP7 is an independent prognostic factor for shorter survival [14], probably because this enzyme can affect tumor cell responsiveness to chemotherapy. MMP7 has been widely studied in cancer progression not only by its implication in ECM degradation and metastasis promotion, but also by its role in the Fas/FasL system regulation and in the apoptosis responsiveness of tumor cells. MMP7 modulates Fas expression and activation, generating the soluble forms of FasL by cleavage of its membrane form [4], and by cleaving Fas receptor itself [8]. In both cases, induction of apoptosis by Fas activation can be blocked by MMP7 activity.

It is well known that morphological and phenotypical characteristics that confer tumor cells advantage against Fas-induced apoptosis are different during tumor progression. In fact, alterations in functional Fas status seem to be produced in parallel to tumor progression towards a more metastatic phenotype [6], [15]. In CRC cell lines different metastatic subpopulations pre-exist within the heterogeneous primary cells. It is known that these cell subpopulations are resistant to Fas-induced apoptosis and that the selective pressure that favours the outgrowth of these metastatic cells selects also for populations with altered Fas functionality [15]. However, loss of Fas expression and functionality during tumor progression is not the unique mechanism accounting for the acquisition of apoptosis resistance. Increased MMP7 expression also contributes to the selection of cells with reduced sensitivity to Fas-induced apoptosis [8], [13], [16], to drug cytotoxicity [4], and to changes in the metastatic phenotype. The apoptotic-effects of some cytotoxic agents such as doxorubicin are mediated by upregulation of Fas and FasL expression, and by activation of the apoptotic signalling in the neighbouring cells [4]. Regarding this, MMP7 can protect cells against death by cleavage of both Fas receptor and its ligand from the cell surface, reducing the effectiveness of FasL in triggering apoptosis [4], [8], [13], [16].

In the present work, we demonstrate that MMP7 expression increases when tumor cells acquire resistance to oxaliplatin. The inhibition of MMPs restores cell sensitivity either with a chemical inhibitor or with inhibition of MMP7 by siRNA, in part by increasing the levels of Fas receptor at the cell surface. One mechanism by which oxaliplatin induces cell death is by Fas upregulation, as previously described for other cytotoxic drugs [4]. However, we have observed that in resistant cells the upregulation of Fas expression by oxaliplatin is partially blunted. Moreover, in these cells Fas, which normally activates apoptotic pathways, switches its function and induces the expression of survival proteins.

## Results

### MMP7 is related to the acquisition of resistance to oxaliplatin in colon cancer cells

**Figure 1:**
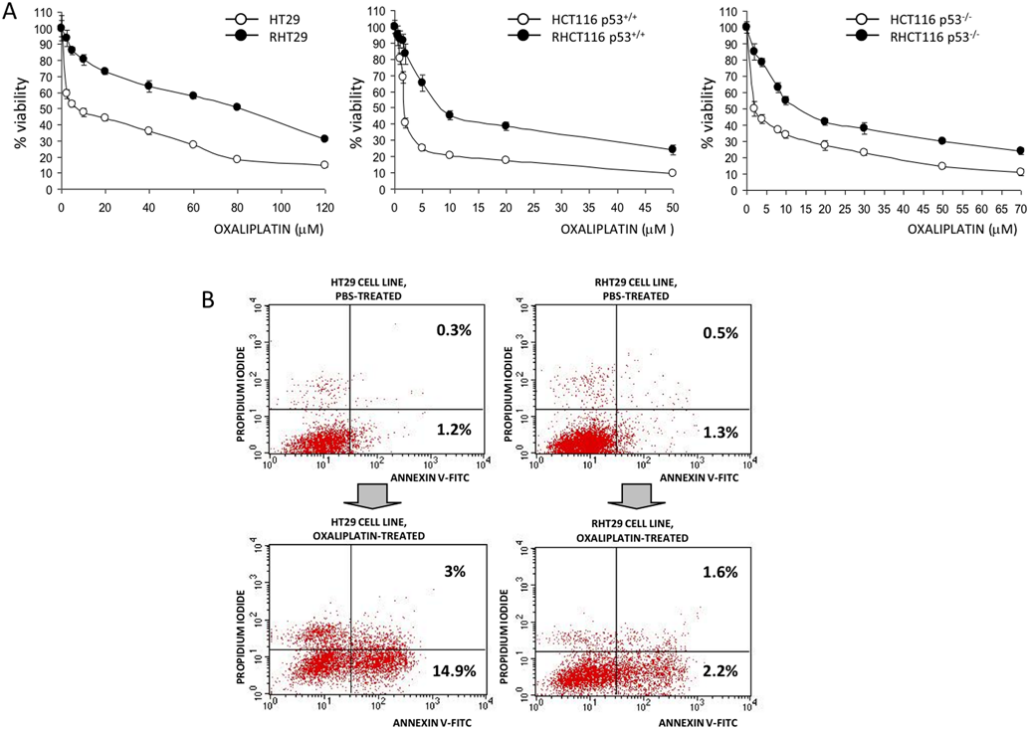
Characterization of cell lines resistant to oxaliplatin-induced cell death. A) Non-resistant (HT29, HCT116 p53^+/+^, HCT116 p53^−/−^) and resistant cells (RHT29, RHCT116 p53^+/+^, RHCT116 p53^−/−^) were assessed by MTS for its response to oxaliplatin treatment. The dose-response curve shows a higher IC50 for the resistant cell lines than for the parental cells as expected. Results are represented as mean±SEM for 6 samples in each group. B) HT29 and RHT29 cell lines were treated or not with oxaliplatin at 10 µM for 48 hours and apoptosis induction was detected by Annexin V/IP staining. These are representative graphics of 3 samples per group, showing less apoptotic induction in the RHT29 cell line. *OXA*: oxaliplatin.

**Figure 2:**
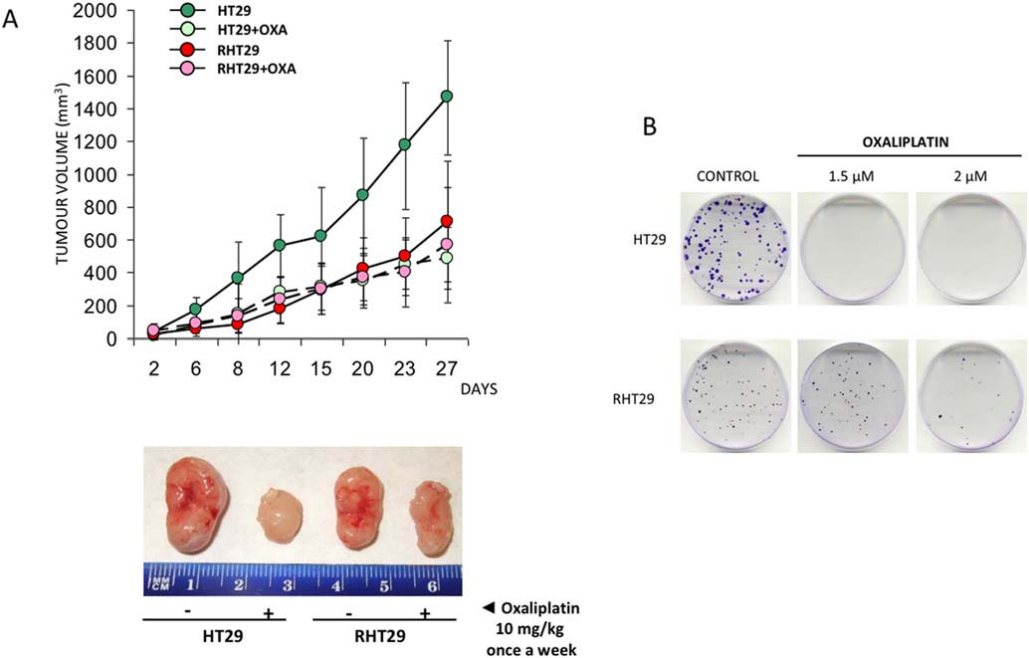
Analysis of HT29 and RHT29 growth rate. A) HT29 and RHT29 cells were subcutaneously inoculated into nude mice. Two weeks later, animals were treated with oxaliplatin 10 mg/kg once a week for 27 days. Results illustrate tumor volume and are represented as mean±SEM for 10 animals per group. The lower panel is a representative image of tumors obtained from animals treated or not with oxaliplatin. B) Growth kinetics was studied by colony formation assay. This representative image shows that HT29 cells are not able to grow under oxaliplatin treatment compared to RHT29 cell line. However, HT29 cells have a higher clonogenic capacity than RHT29 after 21 days of growth. *OXA*: oxaliplatin.

Some authors have observed that addition of MMP7 to cell cultures protects cells from drug cytotoxicity [4] and from cytotoxic T-cell killing [8], but whether MMP7 exerts these effects physiologically or if its expression is related to the acquisition of drug resistance remains unresolved. To analyze the role of MMP7 in these processes, we first generated cell lines resistant to oxaliplatin treatment by exposure of the parental cells to the drug over 5 months. These oxaliplatin-resistant cells show an IC50 several fold higher (RHT29 80 µM, RHCT116 p53^+/+^ ∼8 µM and ∼15 µM RHCT116 p53^−/−^) than the parental (HT29 ∼5–7 µM, HCT116 p53^+/+^ ∼1.75 µM and ∼2 µM HCT116 p53^−/−^),as determined by MTS assay (Figure 1A), and they are more resistant to oxaliplatin induced apoptosis (Figure 1B), as analyzed by annexin/IP staining (5-fold double staining for HT29 compared to 2-fold double staining for RHT29). We chose the HT29 normal and resistant cells for the *in vivo* experiments since they shown the most resistant phenotype. *In vivo*, RHT29 also shows resistance when the drug is administered as a single dose of 10 mg/kg once a week for 4 weeks (Figure 2A). Moreover, characterization of their growth dynamics and their colony formation properties show that, although resistant cells can grow under drug treatment, its growing rate is much lower compared to non-resistant cells (Figure 2B), in agreement with other studies [17]. This lower growth rate was also observed *in vivo* (Figure 2A). On the other hand, the RHT29 cell line shows phenotypic changes consistent with a more mesenchymal phenotype, as it was observed by increased intercellular separation, spindle-shape morphology and formation of pseudopodia (not shown), as previously described for this model of chemoresistance [17].

**Figure 3:**
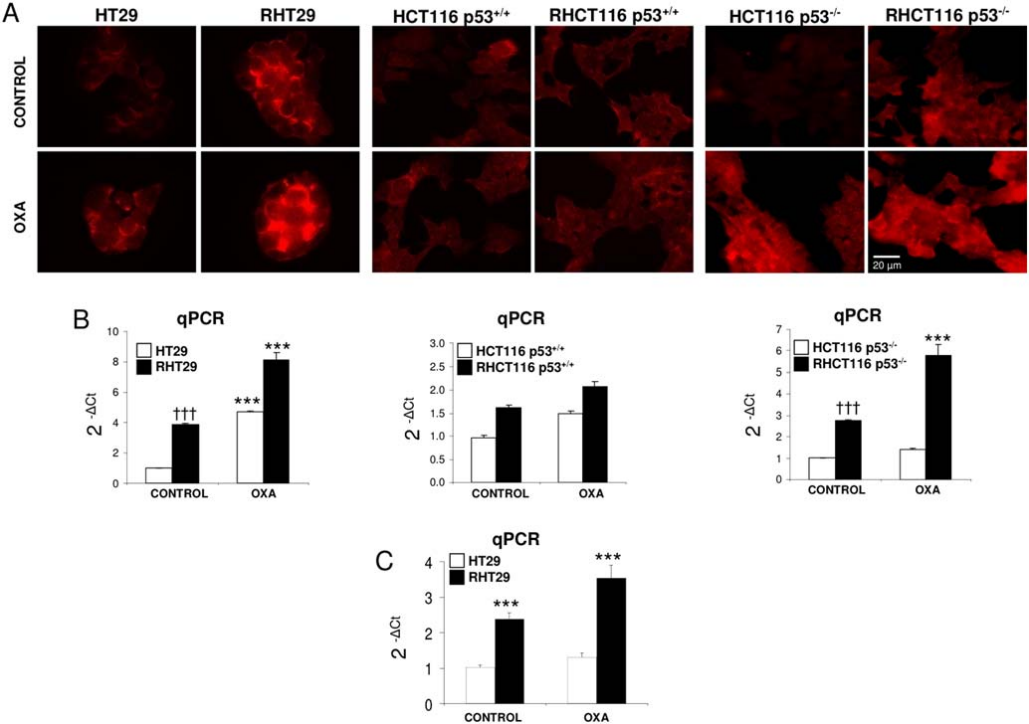
MMP7 shows a higher expression in the oxaliplatin resistant cell lines. A) Immunofluorescence (IF) staining of MMP7 in the non-resistant (HT29, HCT116 p53^+/+^, HCT116 p53^−/−^) and resistant cells (RHT29, RHCT116 p53^+/+^, RHCT116 p53^−/−^). MMP7 is strongly upregulated in the RHT29 and RHCT116 p53^−/−^ cell lines and its expression is also increased by oxaliplatin treatment. Levels of MMP7 were also analyzed by qPCR both *in vitro* (B) and *in vivo* (C) obtaining a similar pattern than in the IF assay. Results represent the mean±SEM for 6 samples in each group. Values that are significantly different between control and treated group by ANOVA analysis are indicated by *p<0.05,**p<0.001,***p<0.0001, and between different cell lines †††p<0.0001. *OXA*: oxaliplatin.

We determined MMP7 expression in these cell lines and we observed that this protease was upregulated in the RHT29 (p53 mutated) and HCT116 p53^−/−^ cell lines but not in HCT116 p53^+/+^ cell line, as we observed by immunocytochemistry and by qPCR (Figure 3A and 3B). In the case of the HT29 and RHT29 cells, we performed an ELISA from the supernatants and found also increased levels of MMP7 secretion in the resistant cells (not shown). Interestingly, it was noticed that treatment with oxaliplatin increases the expression of MMP7 in the RHT29 and HCT116 p53^−/−^ cell lines but not in HCT116 p53^+/+^ cell line (Figure 3A and 3B) and in the xenografts of HT29 and RHT29 cells (Figure 3C), suggesting that p53 status is related to the upregulation of MMP7 expression in these cell lines.

These results demonstrate that MMP7 expression increases during the acquisition of resistance to oxaliplatin in CCR cell lines, and that this protease could also be upregulated by oxaliplatin treatment.

### MMP7 influences the response of tumor cells to oxaliplatin-induced cell death

**Figure 4:**
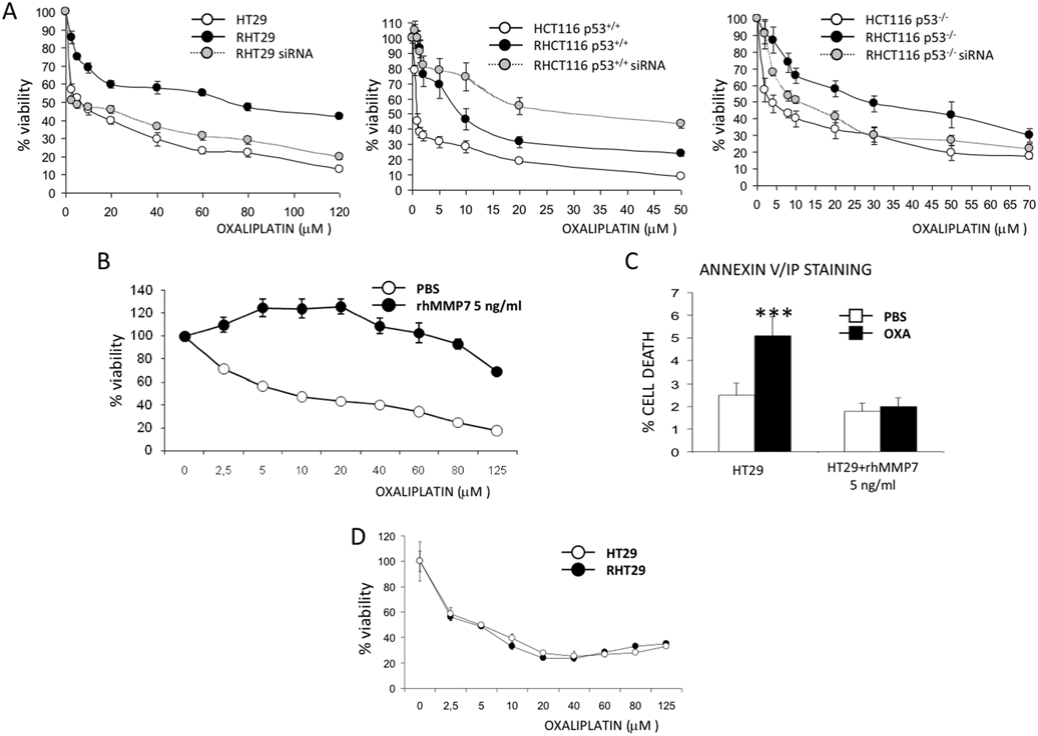
Inhibition of MMP7 by siRNA or addition of rhMMP7 modulates cell response to oxaliplatin-induced apoptosis. A) MMP7 expression was inhibited by siRNA and then treated with different doses of oxaliplatin in order to analyze the effects of MMP7 inhibition in the response to oxaliplatin treatment. Cell viability was then assessed by MTS assay. To analyze the protective effects of MMP7 cells were treated with rhMMP7 (5 ng/ml) and with different doses of oxaliplatin. The effects on cell viability were determined by B) MTS or C) by annexin V/IP staining. D) The effects of MMPs inhibition on cell survival were determined in HT29 and RHT29 cells treated with 1,10-phenantroline monohydrate and then with different doses of oxaliplatin. It can be observed that cells response equally to oxaliplatin when MMPs are inhibited. Results are the mean±SEM for 3 samples per group. Values that are significantly different between control and treated group by ANOVA analysis are indicated by ***p<0.0001. *OXA*: oxaliplatin.

We next investigated whether higher MMP7 expression correlates with loss of cell responsiveness to the drug. For this reason, we inhibit MMP7 by siRNA-directed silencing of this protease. We transfected cells with three different oligonucleotides of siRNA that resulted in decrease of MMP7 mRNA levels by >80% (not shown). Specific inhibition of MMP7 sensitizes the RHT29 and HCT116 p53^−/−^ cell lines to oxaliplatin-induced cell death (Figure 4A), in agreement with previous reports [4]. However, HCT116 p53^+/+^ cell line was not affected by the inhibition of MMP7 (Figure 4A). On the other hand, treatment of HT29 cells with 5 ng/ml of rhMMP7 enzyme also protected cells against oxaliplatin-induced cell death, as we observed in a MTS assay (Figure 4B) and by annexin V/IP staining (Figure 4C).

Because no specific inhibitor of MMP7 exists, we used the MMP inhibitor 1,10-phenantroline monohydrate (referred to hereafter as inhibitor) in additional experiments. Considering that the use of this inhibitor itself may cause cell toxicity, we previously determined by MTS assay and by annexin V/IP staining at which dose we had the lower cell toxicity (not shown). Inhibition of MMPs with 5 µM of 1,10-phenantroline monohydrate restores sensitivity of RHT29 cell line to the drug (Figure 4D).

Therefore, MMP7 is directly related to loss of response to the drug since its inhibition is sufficient to restore the response to the treatment.

### Oxaliplatin fails to overexpress Fas receptor in cells resistant to the drug

**Figure 5:**
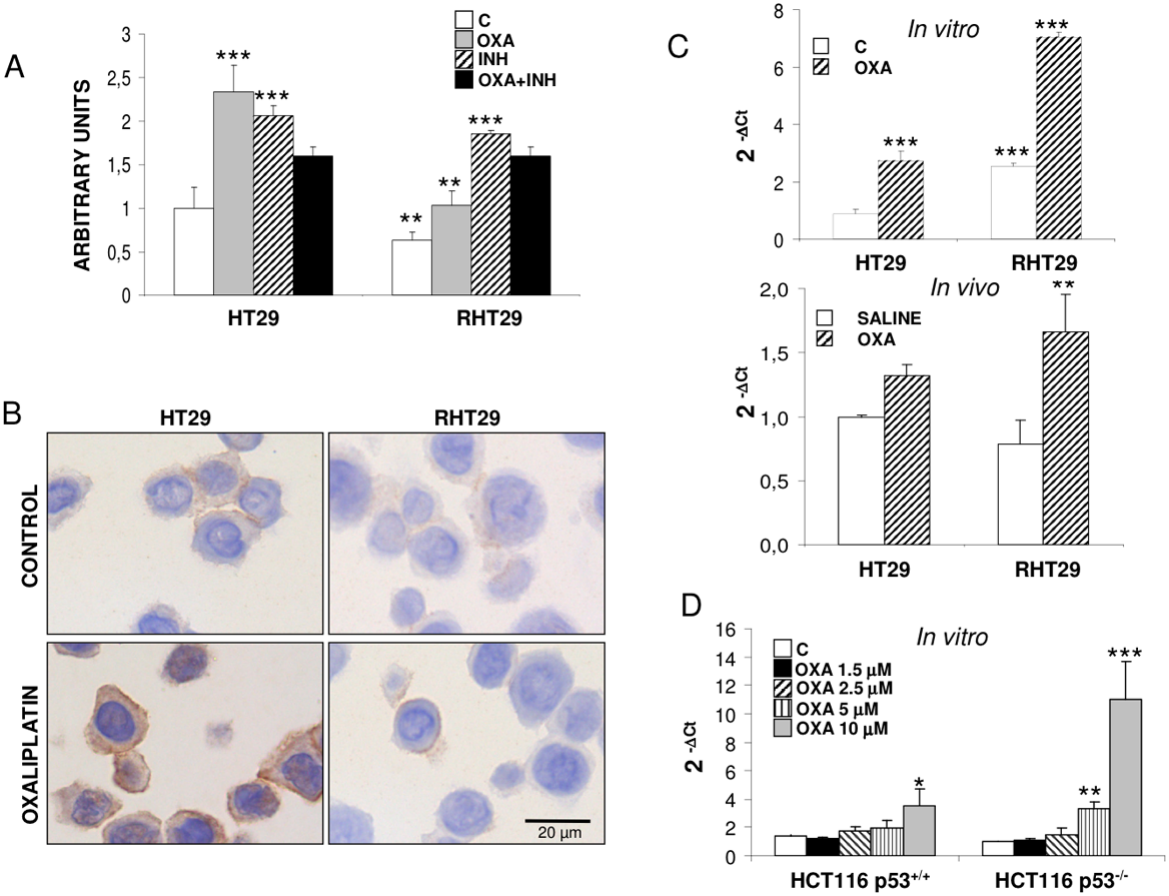
Modulation of cell surface Fas expression in HT29 and RHT29 cell lines. To analyze the expression specifically in the plasma membrane, Fas receptor was determined by A) FACS analysis or B) by immunohistochemistry in a cell line microarray, as described in Materials and Methods. Fas mRNA expression under oxaliplatin treatment was also determined C) *in vitro* and *in vivo* for the HT29 and RHT29 cell lines, D) and also *in vitro* for the HCT116 p53^+/+^ and HCT116p53^−/−^ cell lines. Results are represented as mean±SEM for 5 samples per group in the *in vitro* experiments (C and D) and for 10 animals per group in the *in vivo* determinations (C). Values that are significantly different between control and treated group by ANOVA analysis are indicated by *p<0.05,**p<0.001,***p<0.0001. *INH*: inhibitor (1,10-phenantroline monohydrate), *OXA*: oxaliplatin.

The observation that MMP7 levels increase when cells become resistant to oxaliplatin and that its expression is regulated by the drug, led us to consider that the membrane expression of Fas receptor and its ligand (FasL) was influenced by MMP7 expression. It is well known that Fas and FasL are MMP7 substrates [8], [18], therefore alterations in MMP7 may be directly related to changes in the Fas/FasL system. We decided to measure Fas and FasL expression at the cell surface of HT29 and RHT29 cells by FACS analysis and determine if their expression correlated with MMP7 inhibition. Since MMP7 seems to be the most important protease related to the cleavage of Fas and FasL, we used MMPs inhibitor for the next set of experiments assuming that the effects of this inhibitor in other proteases would be less significant. Regarding Fas expression, oxaliplatin increases the cell surface form of Fas in both cell lines (Figure 5A) similarly to other cytotoxic agents that activate apoptotic pathways by Fas upregulation [5], [19]. However, this increase was significantly lower in the resistant cells (0.6-fold) than in the non-resistant ones (2.3-fold), which suggests that Fas protein levels, as the detected by the DX2 antibody, could be blocked in the RHT29 cell line. To confirm that these alterations in Fas expression were produced at the level of the plasma membrane, we determined the localization of the receptor in cell microarrays of HT29 and RHT29 treated with oxaliplatin for 24 hours. In this case, Fas was also upregulated by oxaliplatin in the normal cells, while resistant cells remained unaltered (Figure 5B). Fas expression at the cell surface can be modulated by MMP7 in a direct way (by cleavage of the receptor) [8] or indirectly by upregulation of soluble forms of FasL which can block Fas [4]. In our case, when MMPs were inhibited by 1,10-phenantroline monohydrate, the levels of Fas at the cell surface were similar in both cell lines (Figure 5A), suggesting that higher levels of MMP7 in RHT29 cells may be responsible for the lower receptor levels in the resistant cells.

To determine if Fas was lower on the cell surface of RHT29 cells because of lower rate of transcription, we determined the mRNA content of Fas in both cell lines cultured with or without the drug. Surprisingly, we observed that oxaliplatin increases mRNA expression of Fas in normal and resistant cells both *in vitro* and *in vivo* (Figure 5C), with a higher degree in RHT29 cells. Although Fas expression has been related to p53 status by several authors [20], [21], these effects were independent of p53 since both cell lines were mutated for the protein. This was confirmed in the HCT116 p53^+/+^ and HCT116 p53^−/−^ cell lines, where we also observed a stronger induction of Fas mRNA by oxaliplatin treatment, mainly in the HCT116 p53-null cells (Figure 5D). These results suggest that, although oxaliplatin upregulates Fas mRNA in HT29 and RHT29 cells in a similar way, the detection of the receptor at the cell surface was lower in the resistant cells because higher MMP7 expression.

### FasL expression is also upregulated by oxaliplatin treatment but decreased in the plasma membrane of resistant cells

**Figure 6:**
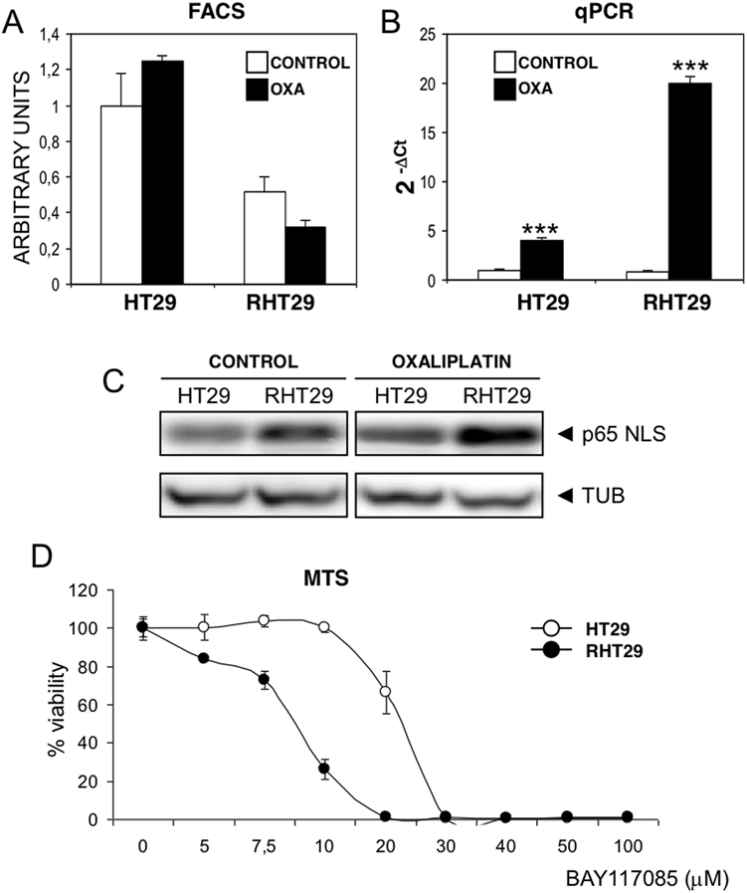
Modulation of FasL expression in HT29 and RHT29 cells lines. FasL expression was detected in HT29 and RHT29 cell lines treated or not with oxaliplatin 10 µM by A) FACS analysis of its surface expression or B) qPCR of its mRNA levels. C) NF-κB p65 was detected by Western Blot by the use of an antibody that recognizes the nuclear localization signal (NLS) epitope. Tubulin expression was used as endogenous control. D) Sensitivity of both cell lines to the inhibition of the NF-κB pathway was measured by an MTS assay in which cells were treated with different doses of BAY117085, a known inhibitor of IKK activation. Values that are significantly different between control and treated group by ANOVA analysis are indicated by ***p<0.0001. *OXA*: oxaliplatin.

We next determined the expression of FasL, a molecule known to be cleaved and released from the cell surface by MMP7 [4]. The analysis of FasL expression under oxaliplatin treatment shows that the drug does not increase significantly the expression of FasL at the cell surface of both cell lines (Figure 6A). However, the mRNA content of FasL was strongly upregulated in both cells lines by oxaliplatin treatment, being these effects more pronounced in the resistant cell line (20-fold) than in the normal one (4-fold) (Figure 6B). It has been observed in other cell lines that cytotoxic treatment could induce FasL expression by upregulation of the NF-κB system. To determine if this system was altered in our model, we decided to analyze the expression of the p65 subunit by using an antibody that recognizes the nuclear localization signal (NLS), an epitope that could be detected when p65 is activated [22]. The expression of NLS-p65 was similar in both cell lines, but under oxaliplatin treatment its activation was higher in the resistant cell line (Figure 6C). Moreover, RHT29 cells showed higher sensitivity to the inhibition of the NF-κB pathway by the IKK inhibitor Bay117085 (Figure 6D) than the non-resistant cells, indicating a metabolic dependence on this pathway by the resistant cell line. Therefore, the increased expression of FasL is correlated with the increased activation of the NF-κB system. These results suggest that, although oxaliplatin treatment induces the synthesis of FasL in both cell lines, its detection at the cell surface is not increased by the drug treatment probably by the shedding of FasL by MMP7.

### Acquisition of resistance to oxaliplatin causes a change in the functionality of Fas receptor

**Figure 7:**
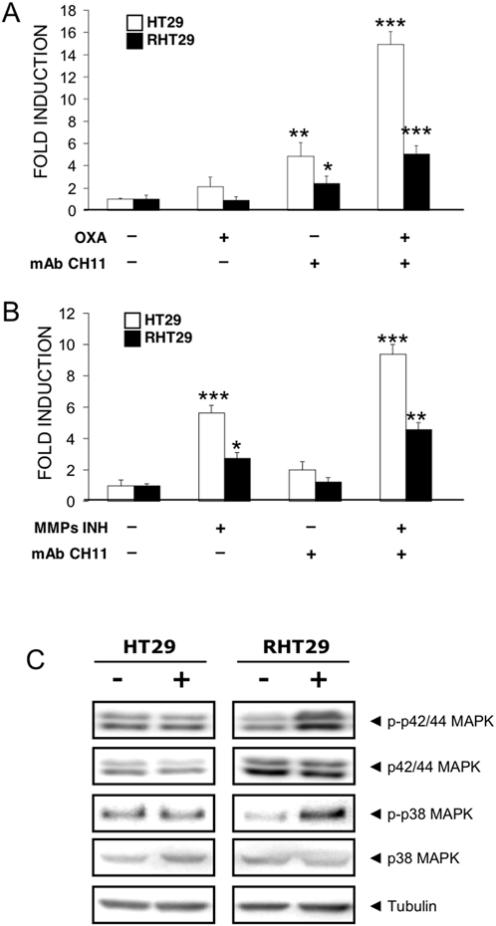
Analysis of the different pathways activated by Fas in HT29 and RHT29 cells. To determine the ability of Fas to induce cell death, cells were treated or not with A) oxaliplatin and B) MMP inhibitor for 24 hours, and then apoptosis was induced by the addition of the agonistic antibody CH11 at a final concentration of 150 ng/ml. Apoptosis induction by Fas activation was determined by annexin V/IP staining as described in Materials and Methods. Results are represented as arbitrary units assuming that control value is 1 and are mean±SEM for 3 samples per group. C) Activation of MAPK pathways in cells treated or not with 150 ng/ml of CH11 for 24 hours was detected by Western blot analysis. Tubulin expression was used as an endogenous control. Values that are significantly different between control and treated group by ANOVA analysis are indicated by *p<0.05,**p<0.001, ***p<0.0001. *OXA*: oxaliplatin.

The lower upregulation of Fas by oxaliplatin at the cell surface of RHT29 led us to consider the possibility of this being one of the mechanisms by which tumor cells avoid drug-induced apoptosis. Besides the levels of expression of the receptor at the cell surface, it has also to be considered its innate function as death receptor. In order to analyze the functionality of the Fas receptor, we determined the induction of apoptosis by the agonistic antibody CH11 when cells were treated with oxaliplatin or with the inhibitor of MMPs. Basal induction of apoptosis by CH11 antibody was undetectable in both cell lines according to previous studies reporting that stimulation of Fas requires previous activation (Figure 7A, 7B). Interestingly, activation of Fas receptor by the agonistic antibody CH11 when cells where treated by both oxaliplatin (Figure 7A) and MMP inhibitor (Figure 7B) was significantly blunted in the resistant cell line (3-fold and 2-fold lower induction for oxaliplatin and MMP inhibitor treatment, respectively), suggesting that in addition to the lower expression of the receptor in RHT29 cells, it is also less potent in activating apoptotic pathways.

Since it has been proposed that Fas can activate other targets and can exert other functions besides apoptosis and, that its activation in some cell lines can have tumor-promoting effects [23], we asked if Fas was inducing survival pathways instead of activating cell death. To address directly this question, we analyzed the levels of phosphorylated and total amount of p42/44 MAPK and p38 MAPK after activation of Fas by CH11 in both cell lines. As it is shown in Figure 7C, activation of Fas by CH11 lead to a higher upregulation of these proteins in the resistant cell line compared to the non-resistant cells, pointing to a change in the functionality of the receptor in cells that have acquired resistance to apoptosis-induction by oxaliplatin.

## Discussion

MMP7 is widely expressed in human cancer [24] and in tumor cell lines [25]. Its expression has been correlated with tumorigenicity [26], with clinical aggressiveness of different tumor types [14], [27], [28], and to loss of response to chemotherapeutic drugs *in vitro* [4], [16]. However, these reports are based on studies aimed at inducing or inhibiting gene expression, which may not reflect the real physiological situation. In this report, we demonstrate that upregulation of MMP7 expression is a direct result of the acquisition of resistance to oxaliplatin, supporting its role in the development of drug-resistance. During the generation of resistant cell lines, only few clones may survive each round of therapy, suggesting that inside the heterogeneous tumor cell population only cells with high expression of MMP7 survive. These results are consistent with the findings reported by others, where it was observed that exogenous addition of MMP7 also increases resistance to drug-induced cytotoxicity [4]. Furthermore, since MMP7 seems to promote tumor survival, it is interesting to note that oxaliplatin treatment also induces MMP7 expression both in normal as well as in resistant cells. This implies a paradoxical effect since a treatment directed to activate apoptotic pathways also induces the expression of proteins that protect cells from death. Moreover, the results obtained with the RHCT116 p53^+/+^ cell line suggest that upregulation of MMP7 is dependent on p53 status.

**Figure 8:**
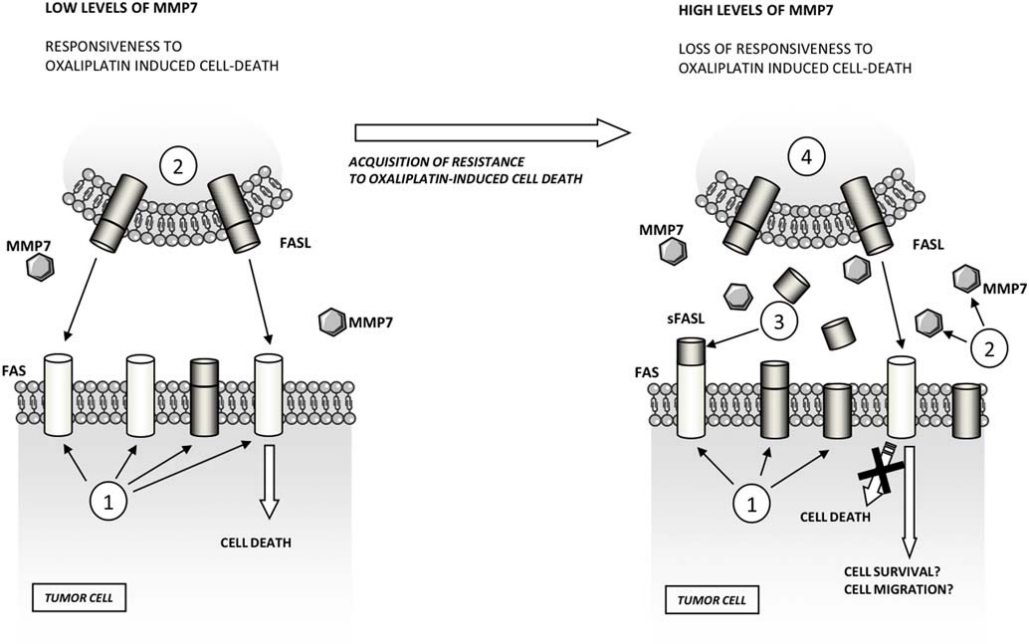
Mechanism of acquisition of resistance to oxaliplatin-induced cell death. A) In a tumor with *low levels of MMP7* oxaliplatin is able to induce an upregulation of Fas receptor at the plasma membrane (1), allowing its activation by cells expressing FasL, such as cytotoxic cells or the surrender cells of the tumor (2). B) On the other hand, in cells with *higher levels of MMP7*, such as cells that have acquired resistance to oxaliplatin, the activation of Fas receptor is different. Oxaliplatin in these cells induce a stronger upregulation of FasL (1) that is shaded by MMP7 to generate sFasL (2). This sFasL binds to Fas probably inducing its activation (3). Fas receptor could, in turn, be shaded by MMP7, decreasing the availability of Fas at the cell surface (4), although the remaining Fas receptor could be activated by its ligand inducing pathways related to cell survival.

One of the mechanisms by which MMP7 protects cells is by favouring the shedding of FasL from cell surface and increasing the proportion of sFasL available to bind Fas [4]. From the results of this work it can be proposed that MMP7 protects cells against Fas-induced cell death by a similar pathway, by cleaving FasL and increasing sFasL, but also by cleaving Fas and decreasing the number of receptors at the plasma membrane. However, it is important to note that during the acquisition of resistance to oxaliplatin a positive loop evolves where the treatment itself upregulates MMP7 (Figure 8). From this point of view, the NF-κB system, which is activated in the oxaliplatin-resistant cells, is also playing a tumor-promoting role regulating FasL synthesis. However, it has to be considered that in these cells Fas is not activating the apoptotic pathways, but promoting pathways related to cell survival and migration. Therefore, it can be suggested that this positive loop generated by oxaliplatin treatment could, at the end, promote tumor progression by Fas activation and induction of tumor-promoting pathways (Figure 8). We are currently testing this hypothesis experimentally.

This relationship between MMP7 and Fas activation could also have implications during tumor progression. The involvement of MMP7 in the promotion of a tumor in its early stages has been discussed by several authors [29], [30]. It has been proposed that MMP7 expression in the first steps of tumorigenesis will select cells with reduced sensitivity to Fas-induced apoptosis [29]. In these stages the sFasL generated by MMP7 could activate apoptosis, allowing the survival of cells that do not activate apoptosis via Fas [4]. These selective effects of MMP7 during tumor progression are similar to those observed during the acquisition of chemoresistance. Therefore, the mechanism of oxaliplatin selection could be mediated by a similar pathway, favouring the selection of cells where Fas activation does not induce apoptosis.

The changes in Fas functionality could have implications regarding FasL functionality. The role of sFasL as a pro- or antiapoptotic stimulus has been largely controversial, but based on the results obtained by us and others [6] it can be proposed that effects of FasL do not depend on its origin (soluble or membrane form) but in the responsiveness of Fas upon activation. Fas expression decreases during tumor progression [31], [32], [33], and many cancer cells acquire a survival advantage by decreasing its sensitivity to Fas-induced apoptosis [7]. Although the activation of survival pathways by Fas engagement has been previously described [23], this is the first report to our knowledge that demonstrates that acquisition of resistance to a cytotoxic drug also induces the loss of ability of Fas to induce apoptosis. Therefore, FasL effects will depend of the molecular pathways activated by Fas.

During tumor progression cells can acquire survival advantages by decreasing Fas expression [31], [32], [33] and its sensitivity to Fas-induced apoptosis [7]. In fact, Fas activation by FasL in cell lines resistant to apoptosis increases motility and invasiveness [23]. In this work, we have demonstrated, to our knowledge, for the first time that when colon cancer cells acquire resistance to oxaliplatin a change in Fas functionality develops, and a receptor switch occurs: from activation of apoptosis to stimulation of MAPK pathways. Therefore, it can be suggested that once the cells have acquired resistance to oxaliplatin, continuous exposure to the drug may favor cell survival by increasing the expression of MMP7 and by activation of Fas, which is regulating cell survival and migration pathways.

## Materials and Methods

### Cell lines and reagents

Human colon carcinoma cell line HT29 was purchased from American Type Culture Collection (Rockville, MD). HCT116 p53^+/+^ and HCT116 p53^−/−^ were generously given by Dr. Vogelstein. The oxaliplatin-resistant cell lines (RHT29, RHCT116 p53^+/+^, RHCT116 p53^−/−^) were developed by repeated exposure of parental cells to increasing concentrations of oxaliplatin over a 5-month period. Experiments were performed with the resistant population obtained from this process instead of using clones to better mimic the normal process by which a patient develops chemoresistance. During the process, cell mortality was assessed for several days and the few cells that survive were recultured in 2.5-cm diameter dishes until they recovered their growth rate. All cell lines were routinely cultured in McCoy's Medium supplemented with 10% heat inactivated foetal bovine serum, antibiotics (Gibco, Invitrogen), and in the case of the resistant cells, with a minimum concentration of oxaliplatin to ensure the development of a resistant population. The cultures were incubated at 37°C in a humidified 5% CO_2_ atmosphere and the cells were serum starved overnight before experiments.

Antibodies used were mouse monoclonal anti-FasL (clone NOK-1) and mouse monoclonal anti-Fas (clone DX2) both from Pharmingen; mouse monoclonal anti-Fas (clone CH11) from Upstate Biotechnology; mouse monoclonal anti-MMP7 (clone ID2) from Abcam; mouse monoclonal anti-NF-κB (NLS epitope) from Chemicon, rabbit polyclonal antibodies against phospho-p42/44 MAPK, p42/44 MAPK, phospho-p38 MAPK and p38 MAPK, were from Cell Signaling Technology, and mouse monoclonal anti-Tubulin from Sigma. MMP inhibitor 1,10-phenantroline monohydrate was purchased from Sigma, and IKK inhibitor Bay117085 was from Calbiochem. Oxaliplatin was from Sigma. All general reagents were purchased from Sigma, Bio-Rad and Amersham.

### Cell viability assay

Cell viability was assessed in subconfluent cell cultures that were incubated for 72 hours with different treatments: oxaliplatin, 1,10-phenantroline monohydrate or both. In the experiments designed to analyze the effects of rhMMP7 (recombinant human MMP7, Chemicon), cells were pretreated with 5 ng/ml of this protease for 24 hours, and then treated with oxaliplatin at different concentrations for 72 hours. Briefly, cells were seeded in 96-well plates at a density of 5.000 cells/well and allowed to attach overnight. After treatment, cells were monitored daily under microscope and cell viability was determined with a tetrazolium compound by CellTiter 96 Aqueous One Solution Cell Proliferation Assay Kit (MTS, Promega), according to the manufacturer's instructions. For colour development, 20 µl of MTS were added to each well and then the plate was read on a microplate spectrophotometer (Molecular Dynamics) at 490 nm (test wavelength) and 690 nm (reference wavelength). Different doses were assessed in sixtuplicate and every experiment was done in triplicate. Assay values for controls were taken as 100% of viability, and the viability at each treatment point was calculated relative to controls by the formula: (Abs 490 nm at every dose/Abs 490 nm at dose 0)×100%.

### Immunofluorescence Assay

For the detection of MMP7 expression, cells were seeded in coverslips until subconfluence and treated with the IC50 for oxaliplatin obtained in each cell line (5 µM, 1.75 µM and 2 µM for HT29, HCT116 p53^+/+^ and HCT116 p53^−/−^, respectively) for 72 hours. Then, cells were washed twice with cold Hepes Buffered Saline buffer (HBS buffer: 135 mM NaCl, 10 mM KCl, 0.4 mM MgCl_2_, 1 mM CaCl_2_, 10 mM Na-Hepes pH 7.4), fixed for 15 min with 4% paraformaldehyde at 4°C, followed by 6 min in methanol at −20°C. After two washes with HBS, cells were blocked for 20 min with 1% of BSA on cold PBS and then incubated with primary antibody against MMP7 overnight at 4°C. Control samples were incubated with buffer or non-specific purified mouse immunoglobulin G. Then, coverslips were washed twice with HBS buffer and incubated for 1 hour at room temperature with anti-mouse secondary antibody labelled with Alexa 594 (Molecular Probes, Invitrogen). After staining, the coverslips were mounted on glass slides with Mowiol (Calbiochem) and viewed using an inverted epifluorescence microscope (Leica). Image assembly and processing were performed using the Image Processing Leica Confocal Software at the Microscopy Unit of the School of Medicine of the University of Barcelona.

### Paraffin-embedded cell arrays and Fas immunohistochemistry

The paraffin-embedded cell line microarray was generated as previously described [34]. Briefly, after trypsinization cells were fixed in 4% paraformaldehyde, washed with PBS and centrifuged. Then, the cell pellet was mixed with 100 µl of melted agar and after its solidification the agar plugs were processed for paraffin embedding as regular biopsies. The blocks obtained were used to construct the cell line array using a tissue microarrayer (Beecher Instruments) and a 2-mm-gauge needle.

Array sections of two micrometer thickness were placed on slides and used for Fas immunohistochemistry with the monoclonal anti-Fas antibody DX2.

### *In vivo* experiments

Animal experiments were performed in accordance with the regulations of our institution's ethics commission, following the guidelines established by the government of Catalonia, Spain. Twenty female athymic nude mice (Harlan), 4–5 weeks old were used in this study. The mice were bred at the Medical School animal facility laboratory and kept under specific pathogen-free conditions at constant ambient temperature (22–24°C) and humidity (30–50%). The mice were given sterilized food and tap water *ad libitum.*

To produce colon carcinoma xenografts, HT29 and RHT29 cells were grown to 80% confluence and trypsinized. Cell viability was confirmed by trypan blue exclusion. Only suspensions consisting of single cells with >90% viability were used for subcutaneous (s.c.) injection of 2×10^6^ cells in 50 µl of PBS in mouse left flank. Treatment began 2 weeks after inoculation, with mice bearing ∼100-mm^3^ subcutaneous tumors. Groups of mice (each group n = 10) were randomly assigned to receive one of the following treatments: a) Intraperitoneal (i.p.) injection of saline solution once a week (control group), b) administration i.p. of oxaliplatin 10 mg/kg once per week during 27 days. Tumors were measured twice per week with a caliper square and, their volumes calculated by the formula: (a×b^2^)/2, where *a* and *b* are the larger and the smaller diameter, respectively. At the end of treatments animals were sacrificed and the tumors removed, minced, placed into liquid nitrogen and stored at −80°C.

### qRT-PCR Analysis

Total RNA from tumor cells was isolated with Ultraspec (Biotecx) according to the manufacturer's instructions. Approximately 1 µg of total RNA was reverse-transcribed into cDNA using the Archived Kit (Applied Biosystems). Negative controls, including those without RNA and reverse transcriptase, were used to confirm the absence of genomic DNA contamination. Quantitative PCR analysis was done on the ABI PRISM 7900 Sequence Detector System (Applied Biosystems). For determination of gene expression Assay-on-demand (Applied Biosystems) was used (Hs00159163_m1 for MMP7, Hs00181225_m1 for FasL, Hs_00531110_m1 for Fas), which consist of a 6-FAM dye-labeled TaqMan MGB probe and the corresponding unlabeled primers. The PCR reaction mixture consisted of 5 µM of each primer, 10 µl of master mix (TaqMan Universal PCR Master Mix, Applied Biosystems) and 2 µl of cDNA. Transcript levels were normalized to those of beta-actin (Hs99999903_m1), which was used as endogenous control. Each experiment and every determination were done at least in triplicate, and the levels of MMP7, Fas and FasL were calculated using the ΔΔCt method.

### Transfection with antisense MMP7

Silencing of MMP7 was done by Stealth RNAi technology from Invitrogen, which provides three different chemical modified oligonucleotides against three different regions of the RNA target. Before silencing experiments, the protocol was adjusted to ensure a minimum of 80% of RNA knockdown assessed by qPCR. According to the manufacturer's instructions cells in a 96 multiwell plate were reverse transfected with Lipofectamine RNAiMAX and 20 nM of each stealth, and 24 hours latter cells were treated with different doses of oxaliplatin as mentioned before. Cell viability was determined after 72 hours by MTS assay. For negative controls, cells were treated with lipofectamine alone or with a GC control (Invitrogen).

### Cytometric determinations

#### Determinations of cell surface Fas and FasL expression

For the detection of cell surface Fas and FasL expression, cells were treated with oxaliplatin (10 µM), 1,10-phenantroline monohydrate (5 µM) or both for 72 hours. Then, cells attached to the plate and from the supernatants were washed with PBS, resuspended in blocking buffer (BSA 1%-PBS) and placed on ice for 30 min. Cell surface staining of tumor cells was done with the following primary monoclonal antibodies: anti-Fas mAb (clone DX2) or anti-FasL mAb (clone NOK1) in blocking buffer for 1 h at 4°C. Cells were then washed 3 times with BSA1%-PBS and stained with FITC-conjugated anti-mouse IgG antibody for 1 hour at 4°C. Control cells without staining were use to determine the settings of autofluorescence, and cells stained with secondary antibody were used to ensure the specificity of the primary antibodies. Stained cells were then analyzed on a FACscan flow cytometer using FACSCalibur (BD Biosciences) and data processed by the software CellQuest. Twelve thousand cells were analyzed for each sample, and every experiment was performed in triplicate. Results are expressed in arbitrary units and have been calculated according to the formula: (fluorescence of each treatment/fluorescence of control cells).

#### Annexin V/IP staining

For the characterization of oxaliplatin resistance HT29 and RHT29 cells were seeded in 6-well tissue culture plates, allowed to attach overnight and subsequently cultured in medium containing oxaliplatin (10 µM) for 48 hours. For the analysis of apoptosis induction, cells were seeded as mentioned before and treated with oxaliplatin (10 µM), 1,10-phenantroline monohydrate (5 µM) or both for 24 h. Then, cells were treated with the Fas-activating antibody CH11 for additional 48 h. To determine the protective effects of MMP7 against oxaliplatin-induced apoptosis cells seeded in 6-well tissue culture plates were treated with rhMMP7 5 ng/ml for 24 hours and then treated with oxaliplatin (10 µM) for additional 24 hours. In all studies apoptosis was determined using Annexin-V-FLUOS Staining Kit (Roche), according to the manufacturer's instructions. Briefly, cells from the culture supernatants and those attached to the plate were blocked for 30 min with 1% of albumin and then incubated for 15 min with Annexin V-FITC and propidium iodide (PI). Controls (binding buffer only, PI only, and annexin-V only) were used to set appropriate detector gains, compensation, and quadrant gates in the FACSCalibur flow cytometer. Twenty thousand cells for each sample were analyzed by the CellQuest software. Every experiment was performed in triplicate

### Western blot

The determinations of the total and activated forms of Erk 1/2 and p38 MAPK and the activated form of NF-κB were performed by Western blotting analysis as previously described [35] in cells treated with CH11 (150 ng/ml) for 24 hours. Tubulin expression was used as an endogenous control.

### ELISA

MMP7 was determined using a quantitative solid phase sandwich enzyme-linked immuno sorbent assay (ELISA) (R&D Systems) as previously described [14] and tested in duplicate. The ELISA for MMP7 can detect both the pro- and active forms of recombinant human MMP7. High concentrations of MMP7 were diluted with calibrator, to produce samples with values within the dynamic range of the assay.

### Colony Formation Assay

For colony formation assay, cells were plated at 500 cells/plate in 6 cm diameter cell culture dishes, treated with oxaliplatin (1.5 and 2 µM) and allowed to grow at 37°C. After 21 days, colonies were stained with crystal violet (Sigma) and scanned to obtain representative images. The experiments were performed in triplicate.

### Statistical analysis

Statistical analysis of the results was performed by ANOVA and Kurskal-Wallis analysis. Statistical significance was considered for p values less than 0.05.

## References

[1] Goldberg RM, Sargent DJ, Morton RF, Fuchs CS, Ramanathan RK (2004). A randomized controlled trial of fluorouracil plus leucovorin, irinotecan, and oxaliplatin combinations in patients with previously untreated metastatic colorectal cancer.. J Clin Oncol.

[2] Desoize B, Madoulet C (2002). Particular aspects of platinum compounds used at present in cancer treatment.. Crit Rev Oncol Hematol.

[3] Chen CC, Chen LT, Tsou TC, Pan WY, Kuo CC (2007). Combined modalities of resistance in an oxaliplatin-resistant human gastric cancer cell line with enhanced sensitivity to 5-fluorouracil.. Br J Cancer.

[4] Mitsiades N, Yu WH, Poulaki V, Tsokos M, Stamenkovic I (2001). Matrix metalloproteinase-7-mediated cleavage of Fas ligand protects tumor cells from chemotherapeutic drug cytotoxicity.. Cancer Res.

[5] Micheau O, Solary E, Hammann A, Martin F, Dimanche-Boitrel MT (1997). Sensitization of cancer cells treated with cytotoxic drugs to fas-mediated cytotoxicity.. J Natl Cancer Inst.

[6] Peter ME, Legembre P, Barnhart BC (2005). Does CD95 have tumor promoting activities?. Biochim Biophys Acta.

[7] Khong HT, Restifo NP (2002). Natural selection of tumor variants in the generation of “tumor escape” phenotypes.. Nat Immunol.

[8] Strand S, Vollmer P, van den Abeelen L, Gottfried D, Alla V (2004). Cleavage of CD95 by matrix metalloproteinase-7 induces apoptosis resistance in tumour cells.. Oncogene.

[9] Chambers AF, Matrisian LM (1997). Changing views of the role of matrix metalloproteinases in metastasis.. J Natl Cancer Inst.

[10] Overall CM, Kleifeld O (2006). Tumour microenvironment - opinion: validating matrix metalloproteinases as drug targets and anti-targets for cancer therapy.. Nat Rev Cancer.

[11] Egeblad M, Werb Z (2002). New functions for the matrix metalloproteinases in cancer progression.. Nat Rev Cancer.

[12] Kurokawa S, Arimura Y, Yamamoto H, Adachi Y, Endo T (2005). Tumour matrilysin expression predicts metastatic potential of stage I (pT1) colon and rectal cancers.. Gut.

[13] Wang WS, Chen PM, Wang HS, Liang WY, Su Y (2006). Matrix metalloproteinase-7 increases resistance to Fas-mediated apoptosis and is a poor prognostic factor of patients with colorectal carcinoma.. Carcinogenesis.

[14] Maurel J, Nadal C, Garcia-Albeniz X, Gallego R, Carcereny E (2007). Serum matrix metalloproteinase 7 levels identifies poor prognosis advanced colorectal cancer patients.. Int J Cancer.

[15] Liu K, McDuffie E, Abrams SI (2003). Exposure of human primary colon carcinoma cells to anti-Fas interactions influences the emergence of pre-existing Fas-resistant metastatic subpopulations.. J Immunol.

[16] Fingleton B, Vargo-Gogola T, Crawford HC, Matrisian LM (2001). Matrilysin [MMP-7] expression selects for cells with reduced sensitivity to apoptosis.. Neoplasia.

[17] Yang AD, Fan F, Camp ER, van Buren G, Liu W (2006). Chronic oxaliplatin resistance induces epithelial-to-mesenchymal transition in colorectal cancer cell lines.. Clin Cancer Res.

[18] Vargo-Gogola T, Crawford HC, Fingleton B, Matrisian LM (2002). Identification of novel matrix metalloproteinase-7 (matrilysin) cleavage sites in murine and human Fas ligand.. Arch Biochem Biophys.

[19] Kaufmann SH, Earnshaw WC (2000). Induction of apoptosis by cancer chemotherapy.. Exp Cell Res.

[20] Muller M, Wilder S, Bannasch D, Israeli D, Lehlbach K (1998). p53 activates the CD95 (APO-1/Fas) gene in response to DNA damage by anticancer drugs.. J Exp Med.

[21] Petak I, Tillman DM, Houghton JA (2000). p53 dependence of Fas induction and acute apoptosis in response to 5-fluorouracil-leucovorin in human colon carcinoma cell lines.. Clin Cancer Res.

[22] Kaltschmidt C, Kaltschmidt B, Henkel T, Stockinger H, Baeuerle PA (1995). Selective recognition of the activated form of transcription factor NF-kappa B by a monoclonal antibody.. Biol Chem Hoppe Seyler.

[23] Barnhart BC, Legembre P, Pietras E, Bubici C, Franzoso G (2004). CD95 ligand induces motility and invasiveness of apoptosis-resistant tumor cells.. EMBO J.

[24] Ii M, Yamamoto H, Adachi Y, Maruyama Y, Shinomura Y (2006). Role of matrix metalloproteinase-7 (matrilysin) in human cancer invasion, apoptosis, growth, and angiogenesis.. Exp Biol Med (Maywood).

[25] Giambernardi TA, Grant GM, Taylor GP, Hay RJ, Maher VM (1998). Overview of matrix metalloproteinase expression in cultured human cells.. Matrix Biol.

[26] Witty JP, McDonnell S, Newell KJ, Cannon P, Navre M (1994). Modulation of matrilysin levels in colon carcinoma cell lines affects tumorigenicity in vivo.. Cancer Res.

[27] Yamamoto H, Adachi Y, Itoh F, Iku S, Matsuno K (1999). Association of matrilysin expression with recurrence and poor prognosis in human esophageal squamous cell carcinoma.. Cancer Res.

[28] Yamamoto H, Itoh F, Iku S, Adachi Y, Fukushima H (2001). Expression of matrix metalloproteinases and tissue inhibitors of metalloproteinases in human pancreatic adenocarcinomas: clinicopathologic and prognostic significance of matrilysin expression.. J Clin Oncol.

[29] Vargo-Gogola T, Fingleton B, Crawford HC, Matrisian LM (2002). Matrilysin (matrix metalloproteinase-7) selects for apoptosis-resistant mammary cells in vivo.. Cancer Res.

[30] Rudolph-Owen LA, Chan R, Muller WJ, Matrisian LM (1998). The matrix metalloproteinase matrilysin influences early-stage mammary tumorigenesis.. Cancer Res.

[31] Nonomura N, Nishimura K, Ono Y, Fukui T, Harada Y (2000). Soluble Fas in serum from patients with renal cell carcinoma.. Urology.

[32] Mizutani Y, Hongo F, Sato N, Ogawa O, Yoshida O (2001). Significance of serum soluble Fas ligand in patients with bladder carcinoma.. Cancer.

[33] Osorio LM, Aguilar-Santelises M, De Santiago A, Hachiya T, Mellstedt H (2001). Increased serum levels of soluble Fas in progressive B-CLL.. Eur J Haematol.

[34] Ferrer B, Bermudo R, Thomson T, Nayach I, Soler M (2005). Paraffin-embedded cell line microarray (PECLIMA): development and validation of a high-throughput method for antigen profiling of cell lines.. Pathobiology.

[35] Lee KH, Feig C, Tchikov V, Schickel R, Hallas C (2006). The role of receptor internalization in CD95 signaling.. EMBO J.

